# Supplementary material for: Light Induced Electron-Phonon Scattering Mediated Resistive Switching in Nanostructured Nb Thin Film Superconductor
Source: Sci Rep. 2017 Apr 13;7:881. doi: 10.1038/s41598-017-00976-1 (PMC5429844; doi:10.1038/s41598-017-00976-1)
Supplement: Supplementary file 1 — Light Induced Electron-Phonon Scattering Mediated Resistive Switching in Nanostructured Nb Thin Film Superconductor [file 41598_2017_976_MOESM1_ESM.doc]

Supporting Material

**Light Induced Electron-Phonon Scattering Mediated**

**Resistive Switching in Nanostructured Nb Thin Film**

**Superconductor**

*Shafaq Kazim^1^, Alka Sharma^1,2^, Sachin Yadav^1^, Bikash Gajar^1,2^, Lalit M. Joshi^1,2^, Monu Mishra^2,3^ , Govind Gupta^2,3^ , Sudhir Husale^1,2^, Anurag Gupta^1,2^, Sangeeta Sahoo^1,2*^and V.N. Ojha^1,2^*

*^1^Time & Frequency and Electrical & Electronics Metrology, National Physical Laboratory, Council of Scientific and Industrial Research, Dr. K. S Krishnan Road, New Delhi-110012, India.*

*^2^Academy of Scientific and Innovative Research (AcSIR), National Physical Laboratory, Council of Scientific and Industrial Research, Dr. K. S Krishnan Road, New Delhi-110012, India.*

*^3^Advanced Materials & Devices Division, National Physical Laboratory, Council of Scientific and Industrial Research, Dr. K. S Krishnan Road, New Delhi-110012, India.*

**E-mail: sahoos@nplindia.org*

**Contents in Supporting Material:-**

1. Control experiment to evaluate the role of Si capping layer on the observed NPC
2. Cross-sectional FESEM and XPS study: observation of interface and study the elemental phases of Nb and Si in the Si/Nb bilayer film.
3. Representative 4-probe measurements at room temperature for a similar sample presented in Figure 1 in the main manuscript.
4. Summary of various sample parameters used in this work in a tabular form

**1. Control experiment to evaluate the role of Si capping layer on the observed NPC:**

In order to study the role of Si capping layer used as the protective layer on the devices, we have carried out the optoelectronic measurements on two different NNFs based devices, viz., with and without the capping layer. The AFM topography images representing surface morphology are shown in Figure S1 (a) & (b). The related photo-response with time dependent *ΔI* measurements are displayed in Figure S1 (c) & (d). As evident from the AFM morphology, no significant changes appear in the overall morphology and the grain sizes for both the types. However, we observe an enhanced change-in-current (*ΔI*) for the device having no capping layer. This indicates initially that the capping layer acts as a barrier for the film to get direct exposure to the light and the effects result in reduced *ΔI*. Further to evaluate the role of silicon capping in originating NPC, we have performed similar optoelectronic measurements at room temperature using 532 nm laser and halogen light sources on only *a*-Si films with no contribution from NNFs. In result, we find linear IV characteristics in dark as well as under light conditions and along with the positive photoresponse from Si capping layer as shown in Figure S1 (e) & (f). This confirmed that NPC is originating from the NNF, whereas Si capping layer is acting as protecting layer from the adsorbed contamination from the environment.


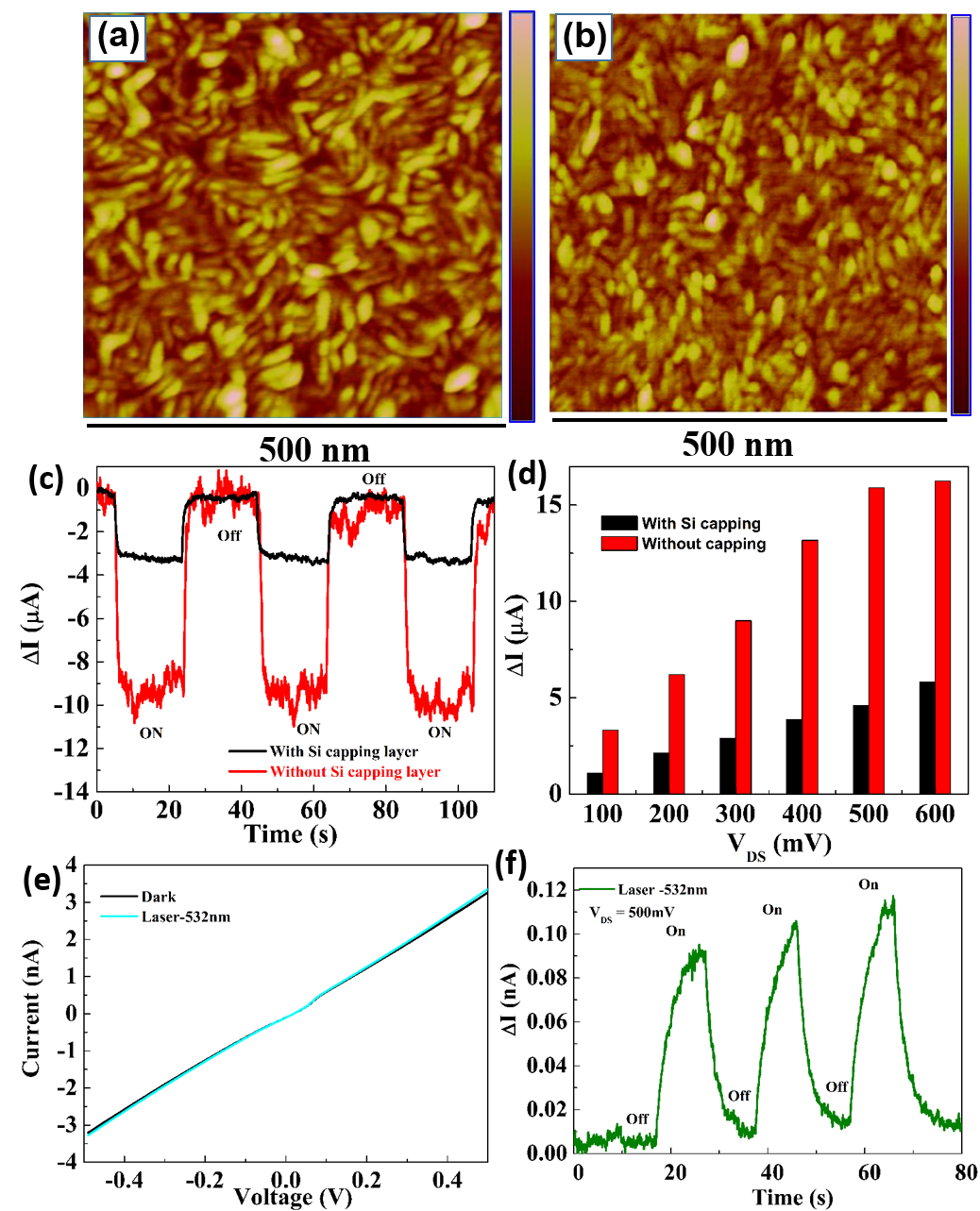


***Figure S1:*** *AFM morphology of NNFs (a) without & (b) with Si capping layer.* *The grains look almost same with grain sizes in the range of 20-30 nm. The colour bars represent the height variations from 0 to 5 nm in (a) and 0 to 6.6 nm in (b). (c) The corresponding real-time measurement of ΔI for light ‘Off’ and ‘On’ cycles. (d) The comparison chart for the bias dependent ΔI for the afore-mentioned two types of devices. (e) IV characteristic for a- Si film having same device geometry under both the conditions. (f) Change in current with time at 500 mV bias voltage for a-Si thin film and shows positive photo-response.*

**2.** **Cross-sectional FESEM and XPS study: observation of interface and study the elemental phases of Nb and Si in the Si/Nb bilayer film:**


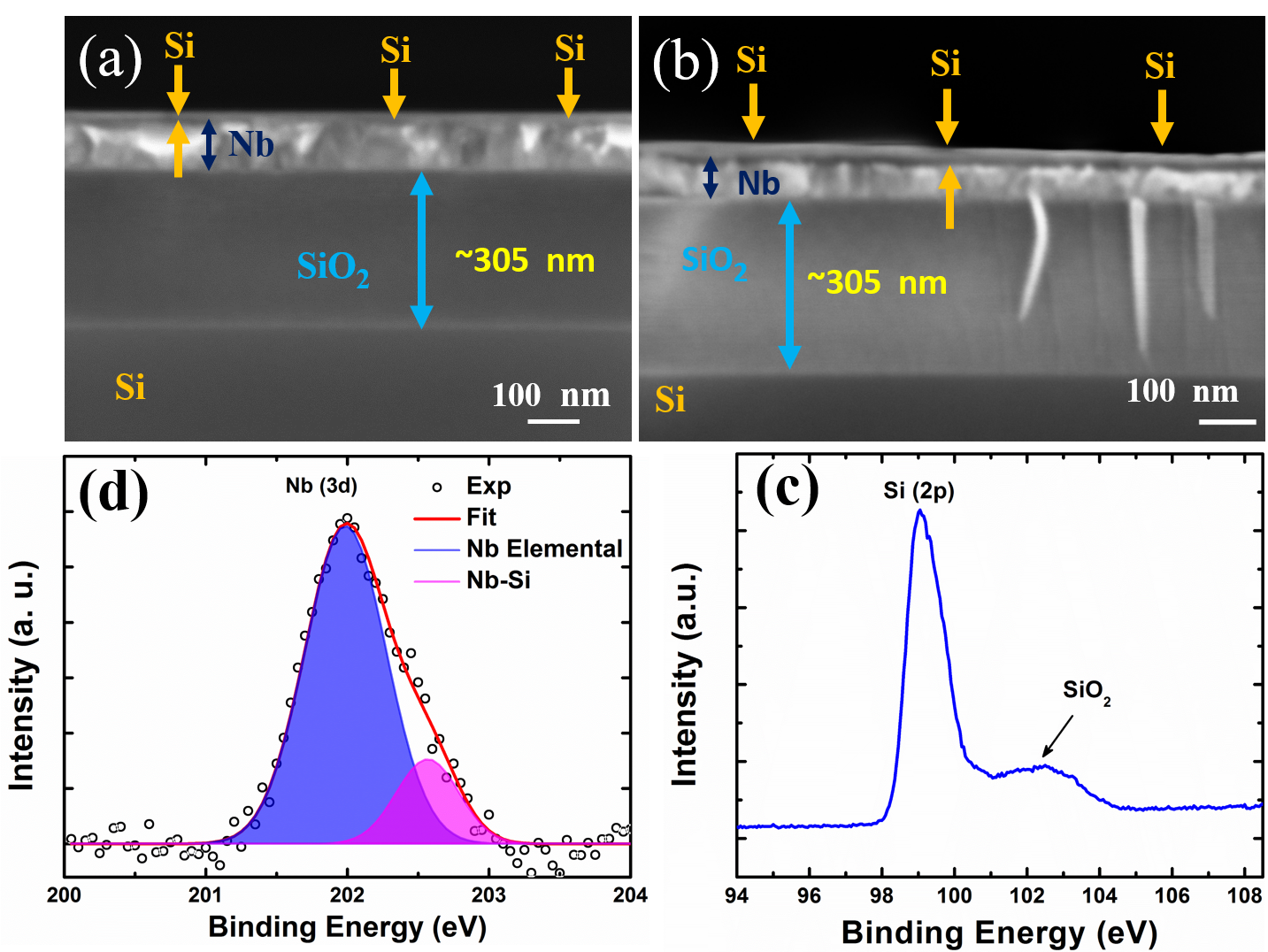


*Figure S2: (a) & (b) FESEM Cross-Sectional view for Nb thin film with Si capping layer on top of it. XPS data presenting (c) Nb 3d and (d) Si 2p spectra for the film with Si capping layer.*

FESEM Cross-sectional view gives a clear indication about the interface between Nb and Si thin film deposited on SiO_2_/Si substrate as marked by different arrows in Figure S2 (a) & (b). For further confirmation, we have performed XPS studies on similar type of thin film samples, and the results are shown in Figure S2 (c) & (d). As there was a capping of Si thin layer on top of the NNFs, soft Ar^+^ ions sputtering (500 eV, 5 minutes) was performed to remove few nanometres of Si capping layer. The binding energy position of Nb 3d_5/2_ appears at 202.0 eV which confirms the dominant presence of elemental Nb. The peak is further de-convoluted to analyse the exact chemical state. It is observed that the Nb 3d_5/2_ peak consists of two components corresponding to elemental Nb (85%) and Nb-Si (15%) with a chemical shift of 0.6 eV^1^. The slight presence of the Nb-Si phase can also have influence on the amplitude of the observed NPC. However, from the XPS results it is evident that the major contribution is from elemental Nb in the studied NNFs. Further, the Si (2p) core level is located at 99.1 eV and reveals the elemental phase of silicon. The SiO_2_ formation is witnessed by the shoulder peak observed at ~103 eV.

^1^ Matthew, J.A.D., Morton, S.A., Walker, C.G.H. and Beamson, G. Auger parameter studies of amorphous NbSi. J. Phys. D: Appl. Phys. **28,** 1702 (1995).

**3. Representative 4-probe measurements at room temperature for a similar sample presented in Figure 1 in the main manuscript:**


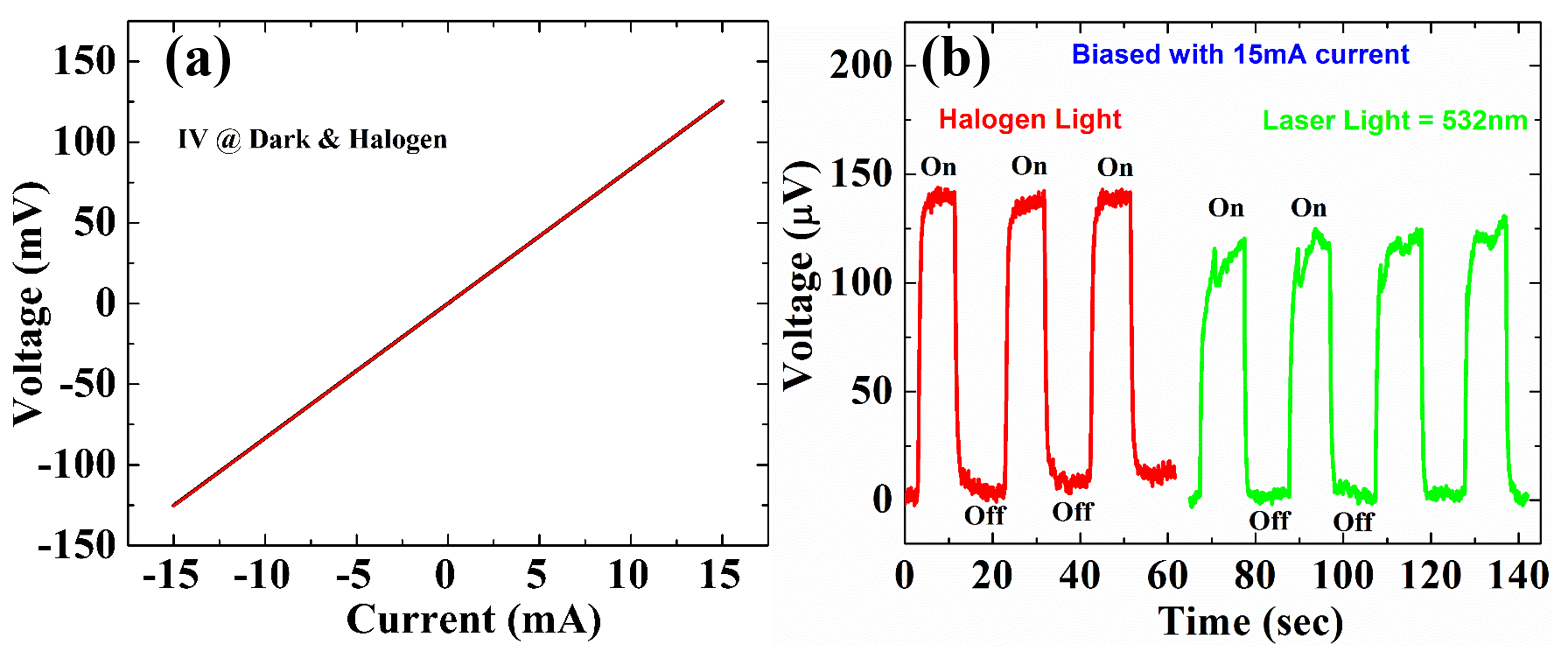


*Figure S3: The room temperature optoelectronic measurements in four probe Geometry: (a) IVC of a NNF device under the dark and illuminated conditions. IVC appears to be linear and follows Ohm’s law. (b) The time dependent voltage characteristics with a constant bias current of 15 mA. The red and green curves correspond the switching behaviour of film with halogen and 532nm laser light respectively.*

In order to compare the device geometry used for performing low temperature measurements on same samples, we have measured the photoresponse by using four-probe measurements at room temperature under halogen and laser light (532 nm) as shown in Figure S3, where current-voltage characteristics (IVC) show linear behavior under dark as well as light conditions and time dependent voltage characteristic biased at 15 mA shows stable switching under light illumination in the four probe measurements and that is similar to two-probe measurements at room temperature.

1. **Summary of various sample parameters used in this work in a tabular form:**

| **Figures related to AFM** | **Growth**  **Condition** | **Thickness**  **(nm)** | **Grain size (nm)** | **ΔI (µA)**  **@ 300mV** | **ΔI (µA)**  **@ 400mV** |
| --- | --- | --- | --- | --- | --- |
| Figure-3(a) | RT grown, No annealing | 100nm | 25nm | 8.99825 | 13.17065 |
| Figure-3(b) | 820°C  Grown and 2hrs annealed | 100nm | 75nm | 12.2816 | 15.675 |
| Figure-3(c) | 820°C  Grown, No annealing | 260nm | 100nm | 27.61 | 50.02053 |
| Figure-3(d) | 820°C  Grown, No annealing | 480nm | 135nm | 33.97771 | 61.26364 |
